# Functional Status and Comorbidities in Older Patient Attending Outpatient in a Tertiary Care Hospital: An Observational Study

**DOI:** 10.31729/jnma.8797

**Published:** 2024-11-30

**Authors:** Ananta Aryal, Anil Aryal, Rhijuta Pokharel, Shrinkhala Timsina, Shirjan Gautam, Saurav Jha, Subash Pant

**Affiliations:** 1 Department of Medicine, Kathmandu Medical College Teaching Hospital, Sinamangal, Kathmandu, Nepal; 2 Nyaya Health Nepal, Achham, Nepal; 3 Kathmandu Medical College Teaching Hospital, Sinamangal, Kathmandu, Nepal

**Keywords:** *comorbidity*, *functional status*, *geriatrics*

## Abstract

**Introduction:**

With the increasing geriatric population, the demand of the geriatric care has been increasing worldwide. Numerous comorbidities like hypertension, COPD, diabetes mellitus, arthritis, are seen commonly in the older population of Nepal, affecting their quality of life. Assessing functional status of individual using Basic Activities of Daily Living and Instrumental Activities of Daily Life scoring is beneficial in predicting the mortality and morbidity among the group. This study hence focuses on determining the comorbidities and functional status of older population.

**Methods:**

This descriptive cross-sectional study was conducted from February 2024 to May 2024 in tertiary care hospital at Kathmandu among population of age group above 60 years, after taking ethical approval from Institutional Review Committee (Reference number: 19012024/04). A total of 423 individuals were taken for the study. Data collection for socio-demographic information, comorbidities, functional assessment via Katz and Lawton scale was done and analysed using IBM SPSS.

**Results:**

Among the total 423 participants visiting the outpatient department, 188 (44.44%) were males and 235 (55.56%) were females. Basic Activity of Daily Living using Katz scoring, suggested that 377 (89.13%; 95% CI: 85.76%-91.93%)) were independent. Using Lawton scoring for the assessment of Instrumental Activities of Daily Living, it was seen that 170 (40.19%; 95% CI: 35.48%-45.03%) were independent. There were 184 (43.50%) individual with comorbidities.

**Conclusions:**

Most of the patients were independent in Basic Activities of Daily Living as per Katz Index of independence and almost half were independent in Instrumental Activities of Daily Living as per Lawton-Brody scale.

## INTRODUCTION

Rising life expectancy has led to growth in the older adult population.^1^ Global elderly population is expected to grow from 1 billion in 2020 to 1.4 billion by 2030.^2^ In South Asia, it is estimated to increase upto 10% of total population by 2025.^3^ According to Nepal's recent census, 10.21% of the population is geriatric.^4^ The World Health Organization (WHO) projects that by 2030, the population over 60 will reach 1.4 billion, increasing the burden of geriatric care and chronic conditions.^5^ The number is set to rise with advances in medicine and technology.^6^

Hypertension, diabetes mellitus (DM), and chronic obstructive pulmonary disease (COPD) are common comorbidities seen among orderly in Nepal.^7^ Independence in Basic Activities of Daily Living (BADL) and Instrumental Activities of Daily Living (IADL) scoring is vital in assessment of functional status of elderly. These indices help to predict a high morbidity and mortality rate in the older population.^8^

This study aims to find prevalence of functional independence by utilizing the Katz and Lawton scale.

## METHODS

This was a descriptive cross-sectional study conducted from February to May 2024 at Kathmandu Medical College Teaching Hospital, a multispecialty hospital in Kathmandu, Nepal. The study population consisted of geriatric patients, defined as individuals aged over 60 years. Comorbidity was defined as the presence of two or more diseases in an individual.^9^ To include the maximum sample the prevalence of functional independence of 50% by both Katz and Lowton scale was assumed. The sample size was calculated by following formula:


$$\begin{array}{ll} \text{n} & ={\text{Z}}^{2}\times \frac{\text{p}\times \text{q}}{{\text{e}}^{\text{2}}}\\ & =1.96^{2}\times \frac{\text{0.50}\times \text{0.50}}{0.05^{2}}\\ & =384 \end{array}$$

Where, Z = 1.96, p = 50% = 0.5, q = 50% = 0.5

Assuming p of study 50%

q = 1-0.5 = 0.5 (q=1-p), allowable error (e) = 5%

The sample size was estimated to be 384 at a 95% confidence level. Adding 10% of the non-response rate, the total sample size is 423. Convenience sampling was used, and all geriatric patients visiting the outpatient department who provided consent were included. All patient aged over 60 years visiting outpatient department (OPD) were included in the study and those not providing or unable to provide consent were excluded from the study.

The Katz Index of Independence in Basic Activities of Daily Living (BADL) was used to assess bathing, dressing, toileting, transferring, continence, and feeding. Katz measures an individual's ability to perform basic self-care independently. A score of 6 indicates independence, 4 indicates moderate dependence, and 0-2 indicates severe dependence.^10^ The Lawton-Brody Instrumental Activities of Daily Living (IADL) scale was used to assess eight higher functions such as using the telephone, shopping, food preparation, housekeeping, laundry, transportation, managing medications, and handling finances. The Lawton IADL scale evaluates higher functioning and an individual's ability to live independently. A score of 8 indicates high functioning, while a score of 0 indicates low functioning.^11^ Body Mass Index (BMI) was classified according to the Asian system of BMI classification.^12^

Ethical approval for the study was obtained from the Ethical Review Committee of Kathmandu Medical College (Reference number: 19012024/04). Written informed consent was obtained from all participants before the study began. Data were collected using a pre-designed form. Basic socio-demographic data, including age, gender, occupation, weight, height, education, marital status, medication use, smoking, alcohol history, physician-diagnosed conditions, and comorbidities were gathered. The questionnaire also included parameters for both Katz and Lawton indices. The data were entered into Excel and analyzed using IBM SPSS Statistics version. Descriptive statistics were used to summarize the data, with a 95% confidence interval applied.

## RESULTS

Among the total 423 participants visiting the outpatient department (OPD), 188 (44.44%) were males and 235 (55.56%) were females. The age range was from 60 years to 96 years with mean age being 69.70 ± 7.66 years among the overall participants and 70.04 ± 7.96 years among males and 69.43 ± 7.40 years among female (Table 1).

**Table 1:** Demographic characteristics of elderly patients (n=423).

**Demographic variables**		Frequency n (%)
Sex	Male	188 (44.44)
	Female	235 (55.56)
Age categories	60-69	223 (52.71)
	70-79	149 (35.22)
	≥ 80	51 (12.07)
Religion	Atheist	1 (0.23)
	Buddhist	20 (4.74)
	Christian	5 (1.18)
	Hindu	396 (93.62)
	Muslim	1 (0.23)
Education level	Illiterate	327 (77.30)
	Primary level	63 (14.89)
	Secondary level	18 (4.26)
	Bachelors	1 (0.24)
	Masters	10 (2.36)
	Post Doctorate	4 (0.95)
Marital status	Married	390 (92.20)
	Unmarried	2 (0.47)
	Divorce	1 (0.24)
	Widowed	30 (7.09)
Living	Alone	24 (5.67)
	With Family	399 (94.33)

A average BMI of the participants was 23.52 ± 4.02 kg/m^2^, with 33 (7.80%) being underweight, 185 (43.74%) normal, 76 (17.97%) overweight at risk, 94 (22.22%) overweight obese I and 35 (8.27%) overweight obese II.

After evaluating the BADL using Katz scoring, it was found that among total participants, 377 (89.13%; 95% CI: 85.76%-91.93%)) were independent (Table 2).

**Table 2:** Katz scoring for BADL in elderly patients (n=423).

Functional status	Frequency (%)
Independent	377 (89.13)
Dependent	46 (10.87)
**If dependent (n=46)**	
Very severe dependence	4 (8.69)
Severe dependence	10 (21.74)
Moderate dependence	32 (69.57)
Total	423 (100.0)

BADL=Basic Activity of Daily Living.

Using Lawton scoring for the assessment of IADL, it was seen that 170 (40.19%; 95% CI: 35.48%-45.03%) were independent (Table 3).

**Table 3:** Lawton Scoring for IADL (n=423).

IADL	Frequency (%)
Independent	170 (40.19)
Dependent	253 (59.81)
Total	423 (100.0)
Severe dependence	10 (21.74)
Moderate dependence	32 (69.57)
Total	423 (100.0)

IALD=Instrumental Activity of Daily Living

There were 82 (19.39%) individuals with no recorded comorbidities, while 184 (43.50%) had comorbidities defined as presence of two or more disease (Table 4).

**Table 4:** Comorbidities distribution in elderly patients (n=423).

**3a. According to presence and absence of morbidities**	
Morbidities	Frequency (%)
One	157 (37.11)
Two or more than two (comorbidity)	184 (43.50)
None	82 (19.39)
**3b. According to the distribution of the individual disease**	
Comorbidities	Frequency (%)
HTN	197 (46.57)
DM	98 (23.17)
COPD	81 (19.15)
Dyslipidemia	59 (13.95)
Thyroid disorder	46 (10.87)
Arthritis	42 (9.93)
APD	40 (9.46)
BPH	25 (5.91)
Heart Failure	14 (3.31)
CLD	11 (2.60)
Dementia	8 (1.89)
Depression	5 (1.18)
Stroke	4 (0.95)
Parkinsonism	3 (0.71)
CKD	2 (0.47)

HTN=Hypertension, DM=Diabetes, COPD=Chronic Obstructive Pulmonary Disease, APD=Acid Peptic Disorder, CLD=Chronic Liver Disease, CKD= Chronic Kidney Disease

## DISCUSSION

In our study, the mean age was found to be 69.70±7.66 years. Among them, the majority of the individuals were living with their family 399 (94.33%) which can be due to cultural tendency of older persons in Nepal living with their children in old age for support.

Under Katz index of functional assessment, our study showed 89.13% independent similar to the study by Sundaram R et. al.^9^ But the value deferred with the data shown by a study by Shrestha KD et. al. (45.00%)^10^ This can be due to the fact that in the mentioned study, the patients were taken from those admitted in the ward. The ward admitted patients relatively require more assistance in their daily activities than those who are visiting the OPD.

From the Lawton index of functional assessment for IADL, more than half of the participants (59.81%) were found to be dependent. One of the reasons for this finding can be attributed to the numbers and types of parameters used to estimate the score. Eight parameters requiring high skills like financial independence are used in the scoring. And considering the literacy rate of Nepal still being below 76.20%,^6^ this fact might have also contributed to the increased dependency on IADL scoring.

Our study showed an increase in comorbidities 184 (43.50%) compared to a study by Balakrishan S et. al. which had comorbidities among 22.80% participants only.^7^ The increase in comorbidities in our study can be linked to the more accessibility to health services, as majority of the participants live in urban area and are being taken care with their family. This can lead to early diagnosis, of the disease in the population.

Hypertension was found to be the most common disease condition in our study, with the overall frequency of 197 (46.57%). The observed freqency in our study was higher than in a study done by Balakrishan S et. al. which reported 31.60% hypertensive in elderly population.^4^ Moreover, hypertension was observed most commonly in the age group above 80 years of age accounting 36 (70.59%) of cases. Similar findings were observed in other studies.^7, 11^ In our study 184 (43.50%) patients had comorbidities, similar findings has been reported in various studies.^12-15^ Soley-Bori et al. synthesizing a systematic review pointed out that multimorbidity's impact was on increased healthcare costs and utilization, therefore putting pressure on the financial resources of the systems.^16^ This is further supported by quantitative study of outlay and usage of older patients with multiple chronic diseases where literature revealed that it facilitated disproportionate resource consumption and monetary implication for such population.^17^

This study has evaluated the data of the tertiary care centre in the capital of the country, so cannot be used to generalise the overall picture of the entire population of the country. We also failed to classify the population based on the individual comorbidities. Also, our study calculates only the presence and absence of an individual comorbidity, but does not give insight on if the comorbidity was seen along with other comorbidities or was present alone. A further detailed data collection which includes information in the individual comorbidity among the participants have to be collected to acquire a more detailed overview of the status.

## CONCLUSIONS

This study suggest that most of the patient were independent in BADL as per Katz Index of independence and almost half of the geriatric population visiting OPD were independent in IADL as per Lawton-Brody scale. Hypertension was the most common condition observed in the population and about half of the patient had comorbidity.
